# Using multi-objective evolutionary algorithms to predict the parameters that determine membrane resonance in a biophysical model of bursting neurons

**DOI:** 10.1186/1471-2202-15-S1-P79

**Published:** 2014-07-21

**Authors:** David Fox, Hua-an Tseng, Horacio G Rotstein, Farzan Nadim

**Affiliations:** 1 Department of Biological Sciences, NJIT-Rutgers University, Newark, NJ 07102, USA; 2 Department of Mathematical Sciences, NJIT, Newark, NJ 07102, USA

## 

Many neurons exhibit membrane potential resonance (MPR), a peak in the membrane impedance amplitude (|*Z*|) in response to oscillatory inputs at nonzero frequency (***f_max_***) [1]. MPR arises from nonlinearity and timescales of voltage-gated currents and may set frequency of network oscillations. Pacemaker PD neurons of the crab pyloric network show MPR whose *f_max_* is correlated with the network frequency (~ 1Hz) [2]. In contrast, the LP follower neuron shows a higher *f_max_* of ~ 1.4 Hz. The impedance profile of biological PD and LP neurons and the model neuron was measured using a logarithmic ZAP function (*f_min_*=0.1 Hz, *f_max_*=4 Hz) in voltage clamp (*V_low_*=-60mV and *V_high_*=-30mV). The *f_max_*in biological PD neurons increases if either *V_low_* or *V_high_* are increased [3], whereas the LP neuron *f_max_* is only sensitive to *V_high_*. Additionally MPR in the PD neurons is sensitive to blockers of *I_Ca_* and *I_h_*. We hypothesize that: (1) many combinations of parameters can produce MPR in PD and LP neurons; (2) The MPR mechanism in LP is distinct from PD.

Experimentally, *I_Ca_*is difficult to measureand therefore a top-down approach is adopted to elucidate the contributions of *I_Ca_*and *I_h_* to MPR in PD and LP. Because resonance depends on the kinetics of *I_Ca_*and *I_h_*, a brute-force sampling of the parameter space is computationally unfeasible and, therefore, we search for model parameters using a genetic algorithm. The biological data were used to constrain the range of leak, *I_Ca_* and *I_h_* parameters in a single-compartment model. The genetic algorithm, NSGA-II [4] was used to optimize the MPR profile and produce a population of optimal models. A sensitivity analysis of MPR attributes on model parameters was done in these models.

The distributions of optimal parameters were tightly constrained for *g_leak_*, *V*_½_Ca_act_, *V*_½_Ca_inact_ and τ__Ca_inact_. Additionally, strong correlations were observed between τ__Ca_act_ and τ__Ca_inact_ (negative), between *V*_½_Ca_act_ and *V*_½_Ca_inact_ and between* g_Ca_*and *V*_½_Ca_act_ (negative). In models with low *I_h_*, *f_max_* correlated strongly with the frequency which *I_Ca_* peaked, which is controlled by τ__Ca_act_ and τ__Ca_inact_. The parameter sensitivities also support the sensitivity to *I_Ca_*time constants, demonstrating potential targets for neuromodulation.

The MOEA was also used to optimize the *f_max_*shifts with *V_low_* and *V_high_* to produce two model groups with properties that correspond to the differences between PD and LP. These results suggest that *f_max_*shift is due to different activation rates of *I_h_* and therefore these two neurons may generate MPR through different mechanisms; a result which we aim to test experimentally.

Many neurons display emergent properties in response to oscillatory inputs, such as amplified responses in certain frequency bands. These properties may be important in shaping coherent network activity. The underlying nonlinearities and time scales that shape specific features of impedance profiles can be used to link sub-threshold dynamics to supra-threshold voltage responses. We have used an MOEA to understand the multiple underlying ionic mechanisms that generate resonance and explained how PD, and not LP, *f_max_*can be adjusted according to different input amplitudes.
